# Functionalization of Biomedical Ti6Al4V via In Situ Alloying by Cu during Laser Powder Bed Fusion Manufacturing

**DOI:** 10.3390/ma10101154

**Published:** 2017-10-03

**Authors:** Pavel Krakhmalev, Igor Yadroitsev, Ina Yadroitsava, Olga de Smidt

**Affiliations:** 1 Department of Engineering and Physics, Karlstad University, 651 88 Karlstad, Sweden; 2 Department of Mechanical and Mechatronic Engineering, Central University of Technology, Free State, Private Bag X20539, Bloemfontein 9300, South Africa; iyadroitsau@cut.ac.za (Ig.Y.); yadroitsava@gmail.com (In.Y.); 3 Centre for Applied Food Security and –Biotechnology (CAFSaB), Central University of Technology, Free State, Private Bag X20539, Bloemfontein 9300, South Africa; odesmidt@cut.ac.za

**Keywords:** Ti6Al4V-Cu alloy, in situ alloying, microstructure and chemical homogeneity, antibacterial properties, implants

## Abstract

The modern medical industry successfully utilizes Laser Powder Bed Fusion (LPBF) to manufacture complex custom implants. Ti6Al4V is one of the most commonly used biocompatible alloys. In surgery practice, infection at the bone–implant interface is one of the key reasons for implant failure. Therefore, advanced implants with biocompatibility and antibacterial properties are required. Modification of Ti alloy with Cu, which in small concentrations is a proven non-toxic antibacterial agent, is an attractive way to manufacture implants with embedded antibacterial functionality. The possibility of achieving alloying in situ, during manufacturing, is a unique option of the LPBF technology. It provides unique opportunities to manufacture customized implant shapes and design new alloys. Nevertheless, optimal process parameters need to be established for the in situ alloyed materials to form dense parts with required mechanical properties. This research is dedicated to an investigation of Ti6Al4V (ELI)-1 at % Cu material, manufactured by LPBF from a mixture of Ti6Al4V (ELI) and pure Cu powders. The effect of process parameters on surface roughness, chemical composition and distribution of Cu was investigated. Chemical homogeneity was discussed in relation to differences in the viscosity and density of molten Cu and Ti6Al4V. Microstructure, mechanical properties, and fracture behavior of as-built 3D samples were analyzed and discussed. Pilot antibacterial functionalization testing of Ti6Al4V (ELI) in situ alloyed with 1 at % Cu showed promising results and notable reduction in the growth of pure cultures of *Escherichia coli* and *Staphylococcus aureus*.

## 1. Introduction

The modern medical industry successfully uses Laser Powder Bed Fusion (LPBF) to manufacture complex parts from biocompatible materials to produce implants using CAD data based on computed tomography scans. The most commonly used biomaterials are stainless steels, cobalt-chrome and titanium alloys. Development of Ti-based materials for medical applications follows several ways. The first way is a development of novel low-moduli β-Ti alloys possessing Young’s modulus closer to the one of bones [1], which can potentially help to reduce a negative stress shielding effect. Another approach is an implementation of cellular structures allowing the tunability of implant elasticity. This way is advantageous due to the flexible control of the implant elasticity and a possibility for the bone to grow through the implant. In this case, modification of the elastic properties of the implant is achieved via topology optimization procedures or using hierarchical structures [2,3]. The third possible way is to design new alloys with added-value functionality, for instance with embedded antibacterial properties, by alloying.

Infection at a bone–implant interface is the most probable reason for implant failure directly after implantation [4]. Subsequently, utilization of materials with embedded antibacterial properties is advantageous as the antibacterial agent could act locally and permanently at the site of infection. For example, coating of the implant interface with materials that have antibacterial properties is a promising approach to prevent occurrence of the infection. Several metals like silver, zinc and copper have shown such antibacterial properties [5]. Therefore, alloying the base material with an antibacterial agent would increase the functionality and antibacterial performances of the implant.

A good example of the latter approach could be the functionalization of an implant with small amounts of Cu. Copper is a proven antibacterial agent and in small amounts is not toxic to the human body [6]. To show the effect of Cu as an antibacterial agent, Jung et al. [7] have electrochemically deposited small amounts of Cu onto Ti discs. After 10 days of exposure to the Cu, *Escherichia coli* (*E. coli*) and *Staphylococcus aureus* (*S. aureus*) bacteria have experienced a dramatic decrease in growth. Antibacterial functionalization of Ti implants by Cu has also been suggested by Shirai et al. [8]. It has been indicated that Cu addition retained the samples’ biocompatibility and inhibited growth of microbes.

In conventional materials, limited miscibility of elements, narrow solubility ranges or a risk of precipitation of undesirable intermetallic phases could limit the possibility to manufacture some alloys. However, the possibility to achieve alloying in situ, during manufacturing, is a unique option of LPBF. This technology combines advantages of powder metallurgy to mix any possible powders with complete melting of powder mixtures, followed by rapid cooling preventing formation of undesirable phases. It provides a unique opportunity to manufacture customized implant shapes, and design new alloys with compositions often impossible for conventional methods.

Selection of manufacturing parameters guaranteeing efficient in situ alloying is a challenge requiring a trade-off. Too high energy input can lead to a keyhole mode resulting in the deep re-melting of a substrate and a formation of pores in the molten pool [9,10]. At low laser power density and high scanning speed, the molten pool has lower temperature, so the viscosity increases, which can lead to the formation of droplets and insufficient mixing [11].

To have successful in situ alloying, the molten metal should have low viscosity, and the molten pool should exist long enough to guarantee as good as possible mixing of the components. At the same time, the conditions should satisfy the operation window, which is required to manufacture high-quality 3D objects [12]. It has been proven experimentally that LPBF is a suitable method for manufacturing of novel alloys from elemental powders using an in situ alloying. Vrancken et al. [13] highlighted the capabilities of LPBF to the processing of a powder mixture of Ti6Al4V (ELI) and 10 wt % Mo. It has also been shown that optimization of the process parameters and an increase in laser power or a re-melting strategy was effective for improving the homogeneity of LPBF Ti/SiC intermetallic materials [14]. Fischer et al. [15] have produced Ti-26Nb alloy by LPBF of a mixture of titanium and niobium elemental powders. These authors also showed that energy input had a significant effect on porosity and homogeneity of the produced part. Dadbakhsh et al. [16] have manufactured an in situ LPBF Al-5 wt % Fe_2_O_3_ alloy and showed that the layer thickness has a strong influence on the microstructural outcome.

This investigation is dedicated to the manufacture and characterization of Ti6Al4V(ELI)-1 at % Cu alloy made by LPBF from a mixture of Ti6Al4V (ELI) and pure Cu powders. In situ alloying during LPBF is challenging since a combination of good chemical homogeneity and high quality of the final 3D object is required. Optimization of formation of the molten pool and single track plays, therefore, a major role in the quality of the manufactured 3D object. In this investigation, optimal process parameters were identified at manufacturing of single tracks and single layers. These parameters then were used to manufacture 3D specimens. Optical and scanning electron microscopy were used to investigate microstructure and fracture surface. Distribution of Cu at the surface of single layer specimens and in the bulk of 3D specimens was studied by means of SEM-EDS. Tensile tests were carried out using mini-samples to compare strength characteristics of alloyed with Cu and unalloyed LPBF TI6Al4V. Pilot antibacterial functionalization testing of Ti6Al4V (ELI) in situ alloyed with 1 at % Cu showed promising results. 

The results showed that the in situ approach is promising for the manufacture of novel advanced Ti alloy with incorporated antibacterial properties. Nevertheless, further optimization of manufacturing parameters and investigations of possible heat treatment are required to manufacture a homogeneous alloy with tailored mechanical properties. Additionally, it is essential to investigate biocompatibility, bioadhesion and corrosion resistance of a new alloy to guarantee innocuous utilization of new alloys in biomedical applications.

## 2. Materials and Methods

Argon atomized spherical powders of Ti6Al4V (ELI) containing 89.26 wt % of Ti, 6.31 wt % of Al, 4.09 wt % of V, 0.12 wt % of O, and Cu of 99.9% purity were used. The 10th, 50th and 90th percentiles of equivalent diameter (weighted by volume) were 12.6 µm, 22.9 µm, 37.0 µm for Ti6Al4V (ELI) powder, and 9.45 µm, 21.9 µm and 37.5 µm for Cu powder respectively. To produce the Ti6Al4V-1 at % Cu (1.38 wt %) powder mixture, the elemental Cu and Ti6Al4V (ELI) powders were mixed for 1 h. Before the laser processing, the powders mixture was dried at 80 °C for 2 h to increase powder flowability.

Specimens were manufactured on the Ti6Al4V substrate by EOSINT M280 machine at laser power 170 W and spot size of 80 µm. In order to determine the effect of a hatch distance, scanning speed and strategy on the surface quality and distribution of elements in the in situ sintered alloy, rectangular single layers with size 4.5 mm × 12 mm were produced. Rescan was done on the same surface without deposition of a new powder layer. Experiments with single tracks of 20 mm in length, and single layers were done using powder layer of 60 µm in thickness, while for 3D samples 30 µm-movement of build platform in Z direction was used.

The surface roughness of the samples was measured perpendicularly to the scanning direction with Surftest SJ-210 (Mitutoyo Corporation, Kawasaki, Japan) according to ISO 1997 standard. To investigate distribution of the alloying elements in the bulk, 3D disc specimens of 10 mm in diameter and 3 mm in height were manufactured. Vickers hardness of the samples was measured at 200 gram-force loading at polished cross-sections. At least 30 measurements for each cross-section were done. Tensile tests were performed with an MTS Criterion Model 43 electric testing machine (MTS Systems Corporation, Eden Prairie, MN, USA) with wedge grips and without extensometer under a constant strain rate of 0.5 mm/min. Specimen geometry and test details have been presented elsewhere [17]. The crosshead displacement–load curves were recalculated to stress-strain curve after measurements of the specimen elongation after failure. Prior to tests, specimen dimensions were measured and an initial gauge length was marked on specimens, the final gauge length was measured after the test to estimate elongation at failure. Effective load-bearing cross-sections were calculated according to the correction procedure suggested in [18].

Determination of chemical composition at the surface and 3D specimens was done by JEOL JSM-6610 scanning electron microscope with Thermo Scientific Ultra-dry energy-dispersive spectrometer (JEOL Ltd., Tokyo, Japan) and LEO 1350 FEG-SEM (Carl Zeiss Microscopy GmbH, Germany) equipped with Oxford Instruments INCAx-sight EDS detector (Oxford Instruments plc, Abingdon, UK).

To study the effect of Cu addition on the bacterial growth inhibition, a number of Ti6Al4V and Ti6Al4V (ELI)-1 at % Cu discs of 10 mm in diameter and 3 mm in height were manufactured. Antibacterial activity was tested against *E. coli* ATCC 10536 and *S. aureus* ATCC 25923 [8,19,20,21,22] as pure cultures. Each strain was revived from cryo-preservation by plating on nutrient agar and incubating at 37 °C for 24 h. Inoculums of actively growing cultures suspensions were obtained by sub-culturing in nutrient broth and dilution to 10^5^ CFU/ml. These suspensions were used to inoculate the Ti6Al4V and Ti6Al4V (ELI)-1 at % Cu discs. 

Three discs of each manufactured series were subjected to the same bacterial treatment. Each well was inoculated with 800 µl of bacterial suspensions containing 10^5^ CFU/well. Broth with and without a disc, as well as bacterial inoculums without a disc were included as controls. Plates were incubated at 37 °C for 24 h without aeration, gently mixed and the planktonic growth determined. Each disc was rinsed with the bacterial suspension in its well, where after the entire liquid content of each well was removed and diluted for bacterial enumeration. Dilutions were plated on nutrient agar plates using the easySpiral Pro^®^ automatic plater (Interscience, Saint-Nom-la-Bretèche, France). Following incubation for 18–24 h at 37 °C, the number of cultivable bacteria was determined with the Scan^®^1200 Automatic HD colony counter (Interscience, Saint-Nom-la-Bretèche, France) and expressed as CFU/ml. The significance of growth inhibition by Cu in comparison to Ti6Al4V control discs was analyzed using the t-test, with results considered significant if *p* < 0.05.

## 3. Results and discussion

### 3.1. Development of Process Parameters

Development of the process parameters was done according to the hierarchical approach aiming to build up a high-quality product. The approach involves analysis of single tracks, single layers and then 3D objects with a number of feedbacks and responses to optimize the process parameters and improve the final quality of the object [12].

Regular single tracks are the basic building blocks in the LPBF object. Optimization of the formation of the molten pool and single track plays a major role in the quality and mechanical properties of the manufactured 3D object, as the molten pool has to be stable. The geometric characteristics of the LPBF tracks are mainly determined by the feedstock material properties, energy input (laser power, spot size and scanning speed) and the thickness of the deposited powder layer [9]. In the present investigation it was found that at the laser spot size of 80 µm and laser power of 170 W, stable tracks were formed at scanning speed range of 0.7–1.3 m/s. Therefore, further manufacture of single layers and 3D specimens was done at these process parameters.

For 3D LPBF parts, the highest powder layer thickness is a combination of a displacement of the build platform in the Z-direction, surface roughness and shrinkage of the previously processed layer. Obviously, to manufacture high-quality and pore-free objects, a powder layer and a part of the substrate should be completely remolten. To see an influence of the layer thickness on the final quality of the deposited layer, and to make sure that selected manufacturing parameters guarantee good metallurgical bonding with the substrate and the previous layer, a set of single layers was produced. The layers were manufactured at 170 W laser power, 1.0–1.3 m/s scanning speed and 70–100 µm hatch distance.

**Figure 1:**
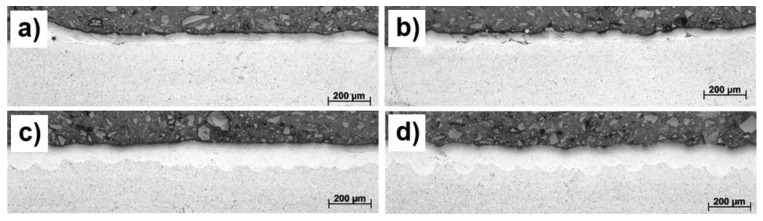
Cross-sections of the surfaces of LPBF Ti6Al4V (ELI)-1 at % Cu manufactured at the laser power of 170 W, scanning speed 1.0 m/s: (**a**) 70 µm hatch distance, single scan strategy; (**b**) 70 µm hatch distance, rescan strategy; (**c**) 100 µm hatch distance, single scan strategy; (**d**) 100 µm hatch distance, rescan strategy.

Investigation of cross-sections of single layers showed that the average remelted depth was about 60–70 µm. After the rescan, the total remelted depth increased to approximately 110 µm, Figure 1. Such a depth was enough to provide good metallurgical bonding between layers. Defects like porosity, delamination of layer or solidification cracks were not observed in the investigated single layers at both, the single scan and the rescan manufacturing strategies applied. It confirmed that the suggested set of manufacturing parameters was optimal to guarantee the metallurgical bonding and defect-free manufacture of the Ti6Al4V (ELI)-1 at % Cu powders, hence it was used for 3D objects.

### 3.2. Optimization of Surface Roughness

While the laser power and scanning speed have a major influence on the geometry of the single track, the hatch distance directly affects surface quality. Hatch distance refers to the distance between centers of two adjacent single tracks. Due to the denudation effect, the first track is wider than the next one [11]. If overlapping of the adjacent tracks in not sufficient, surface roughness is increased due to the formation of deep valleys between tracks. It could result in an increased porosity. Optimal hatch distances are also essential for reduction of the process time and control of thermal cycling during LPBF.

An influence of the manufacturing parameters on the surface quality of Ti6Al4V (ELI)-1 at % Cu was investigated. Results of the investigations showed that the surface roughness increased with an increase in the hatch distance and scanning speed, Table 1. Measured surface roughness values reflect a combination of depth of valleys and heights of peaks at the surface. A height of satellites that were formed from the liquid droplets spluttered from the molten pool during manufacture is an additional contribution in the observed surface roughness values. If specimens were manufactured at higher scanning speeds and higher hatch distances, more satellites were observed on the surface. It led to a dramatic increase of the measured surface roughness parameters. Application of the rescan strategy removed the majority of satellites from the surface, therefore higher surface quality was observed after application of the rescan manufacturing strategy.

Surface quality is usually characterized with *R_a_* value, which is the arithmetic average of the absolute values of the profile height deviations from the mean line, recorded within the evaluation length [23]. Nevertheless, applicability of this value for LPBF surface assessment is arguable. Every surface defect could be an origin of the bulk defect detrimentally influencing quality of the final object. Therefore, it seems logical to use those values of surface roughness that describe extremes in heights or depths. For example, *R_z_* value, which is an average of the vertical distance between the highest and lowest points of the profile within a sampling length [23] could be used. Table 1 shows *R_a_* and *R_z_* values of the investigated surfaces of Ti6Al4V-1 at % Cu. It is seen that the difference between them is in range of an order of magnitude, and for the high hatch distances and high laser scanning speed values, *R_z_* reached about a half of the powder layer thickness. Nevertheless, it is suggested that *R_z_* parameters is more suitable for the assessment of surface roughness of LPBF objects.

### 3.3. Distribution of Cu at the Surface

Since Cu introduces antibacterial properties to the bone–implant interface, a sufficient concentration and distribution of Cu in the matrix alloy is required. It has been shown by Ren et al. [20] that 1–5 wt % Cu added to Ti6Al4V alloy had an obvious antibacterial effect with good corrosion resistance and cytocompatibility. 

**Figure 2:**
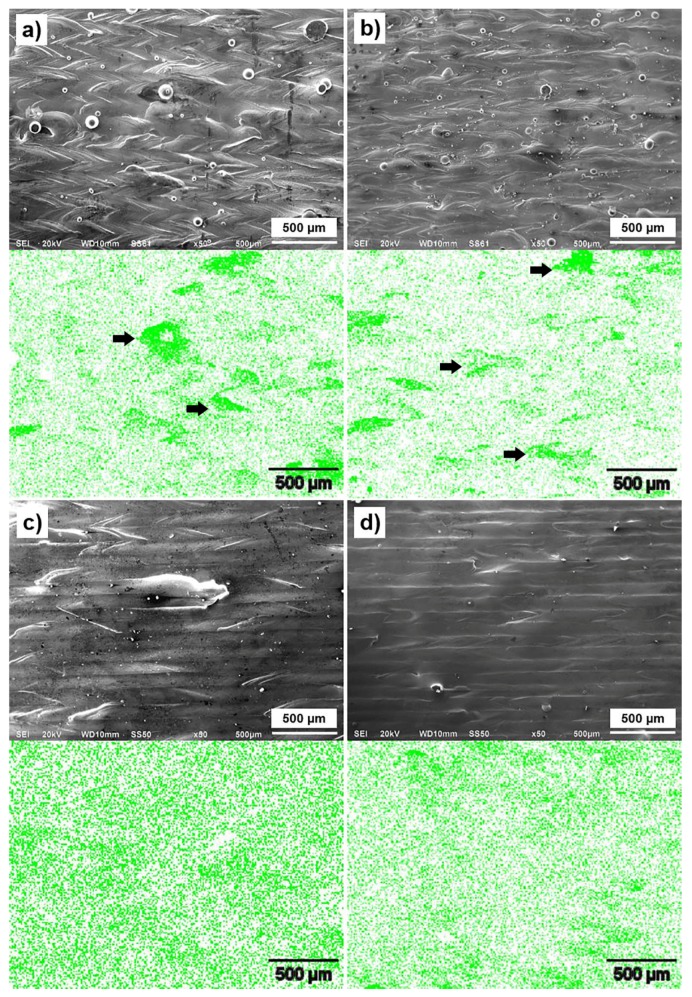
Top view of single layers, and CuK_α_ SEM-EDS maps obtained from the surfaces of LPBF Ti6Al4V (ELI)-1 at % Cu manufactured at laser power of 170 W and 90 µm hatch distance: (**a**) single scan strategy, scanning speed 0.7 m/s; (**b**) single scan strategy, scanning speed 1.3 m/s; (**c**) rescan strategy, scanning speed 0.7 m/s; (**d**) rescan strategy, scanning speed 1.3 m/s. Some Cu islands pointed out with arrows.

In the present investigation, assessment of the homogeneity of Cu in Ti6Al4V at the surface at various scanning speeds (0.7–1.3 m/s) and hatch distances (70–100 µm) was performed by analysis of EDS elemental maps. It was observed that Ti6Al4V-1 at % Cu material manufactured using the single scan strategy, copper particles melted and formed islands following solidification lines at the surface, Figure 2. Concentration of Cu detected by SEM-EDS at the top surface showed in Table 1. Using the single scan strategy, at 0.7 m/s scanning speed and 70 μm hatch distance, concentration of Cu at the surface was 1.05 wt %. Content of Cu increased to 2.31 wt % at scanning speed of 1.3 m/s and hatch distance of 100 μm. Generally, for both, the single scan and the rescan manufacturing strategies, with an increase in scanning speed and hatch distance concentration of Cu at the surface increased.

A change in a scanning strategy from the single scan to the rescan removed a majority of the copper segregations that formed at peripheries of the single tracks, Figure 2. It could be due to the repeating complete re-melting of the material. Nevertheless, similar to the results observed for the single scan strategy, at the rescan the Cu inhomogeneity slightly increased with a decrease in energy input, i.e., with an increase in scanning speed and hatch distance. After an application of the rescan strategy, the closest to a nominal concentration of Cu was observed at 0.7–0.9 m/s scanning speed, at hatch distances of 70–90 µm.

In the present investigation, assessment of the homogeneity of Cu in Ti6Al4V at the surface at various scanning speed (0.7–1.3 m/s) and hatch distances (70–100 µm) was performed by analysis of EDS elemental maps. It was observed that in Ti6Al4V-1 at % Cu material manufactured using the single scan strategy, copper particles melted and formed islands following solidification lines at the surface, Figure 2. Concentration of Cu detected by SEM-EDS at the top surface showed in Table 1. Using the single scan strategy, at 0.7 m/s scanning speed and 70 μm hatch distance, concentration of Cu at the surface was 1.05 wt %. Content of Cu increased to 2.31 wt % at scanning speed of 1.3 m/s and hatch distance of 100 μm. Generally, for both, the single scan and the rescan manufacturing strategies, with an increase in scanning speed and hatch distance concentration of Cu at the surface increased.

A change in a scanning strategy from the single scan to the rescan removed a majority of the copper segregations that formed at peripheries of the single tracks, Figure 2. It could be due to the repeating complete re-melting of the material. Nevertheless, similarly to the results observed for the single scan strategy, at the rescan the Cu inhomogeneity slightly increased with a decrease in energy input, i.e., with an increase in scanning speed and hatch distance. After an application of the rescan strategy, the closest to a nominal concentration of Cu was observed at 0.7–0.9 m/s scanning speed, at hatch distances of 70–90 µm. 

### 3.4. Distribution of Cu in the Bulk

**Figure 3:**
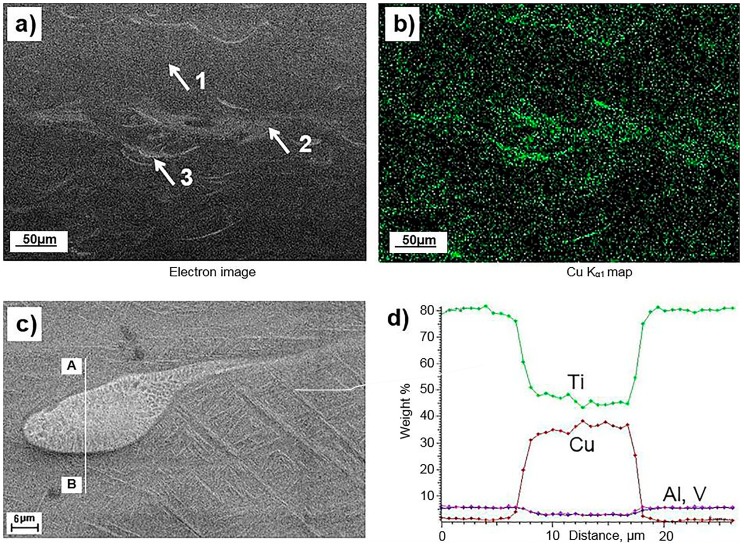
Cu-enriched regions in LPBF Ti6Al4V (ELI)-1 at % Cu alloy: (**a**) back scattered electron image; (**b**) CuK_α_ SEM-EDS map of a cross-section made along building direction; (**c**) Cu-rich droplet-like area and (**d**) elemental concentration profiles in wt % of Ti, Al, V and Cu in the Cu-rich area illustrated in (**c**) obtained along line AB. Arrows indicate typical microstructures, 1—regular grey martensitic, 2—bright-gray martensitic, 3—bright droplet-like regions.

Distribution of Cu in the bulk LPBF Ti6Al4V (ELI)-1 at % Cu material was studied with SEM-EDS on cross-sections of discs. Investigations have shown that Cu was quite well dissolved in the Ti alloy matrix, although some areas enriched with Cu were observed. Figure 3a,b illustrate distribution of Cu in the bulk material.

**Figure 4:**
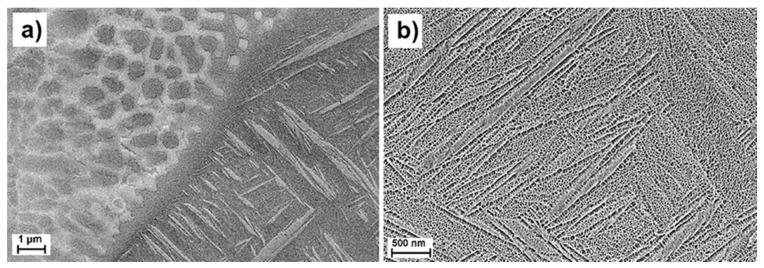
Microstructure of LPBF Ti6Al4V (ELI)-1 at % Cu alloy, (**a**) dendritic/cellular area with higher Cu content adjacent to martensitic region and (**b**) purely martensitic structure with lower Cu content.

Observed Cu-rich areas had characteristic shapes, which could easily be associated with the molten pool boundaries, Figure 3. Microstructure in these areas also differed from the regular surrounding structure. Regular microstructure of the matrix, visible as dark grey regions, depicted as region 1 in Figure 3a, had typical for α′-hexagonal martensite needle-like structure, commonly observed in unalloyed Ti6Al4V manufactured by LPBF [24,25]. Brighter regions (2 and 3 in Figure 3a) had two different morphologies. In regions 2, visible like light-grey regions decorating molten pool boundaries, microstructure also had needle-like morphology typical for martensitic phase. In these areas, the observed needles were apparently coarser compared to the ones observed in region 1, Figure 4. Regions 3 are the brightest in Figure 3a. These areas often had a droplet-like shape and rather cellular/dendritic microstructure, as seen in Figure 3c. 

EDS analysis of the regions 1, 2 and 3 showed that the differences in the microstructure correlate with concentration of Cu. Regions 1 in Figure 3a, although had regular martensitic structure, were not pure Ti6Al4V, but material alloyed with Cu. Copper content in these areas achieved 1–1.5 wt %, no regions of pure Ti6Al4V were observed. In the brighter regions 2, SEM-EDS showed higher concentration of Cu comparing to the regular surrounding material. These regions still had martensitic structure, but Cu content varied in range 2–5 wt %. The highest concentration of Cu, up to 35 wt %, was observed in regions 3 that have dendritic/cellular microstructure. The observed Cu-rich areas were not pure Cu, as concentration of Ti in those areas was high, Figure 3d. No pure Cu particles were detected in the microstructure by SEM-EDS.

**Figure 5:**
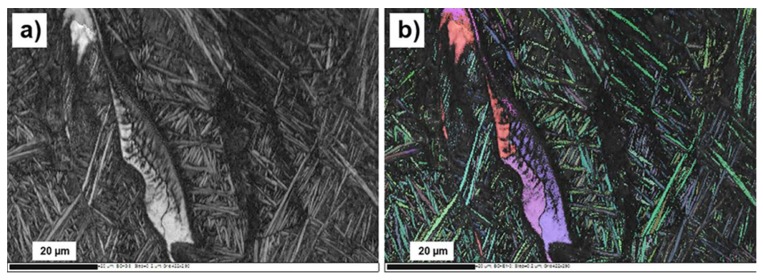
EBSD image of LPBF Ti6Al4V (ELI)-1 at % Cu, droplet-shaped area with high concentration of Cu: (**a**) channeling contrast image; (**b**) EBSD map, shades of green color correspond to areas with hexagonal crystal lattice, areas of red-purple color have cubic crystal lattice.

Electron backscatter diffraction (EBSD) investigations confirmed that the areas with concentration of Cu 1–5 wt % had Ti-hexagonal structure, while in areas where concentration reaches 30 wt % Cu and higher, Ti-cubic structure was observed. Figure 5 illustrates a Cu-enriched area surrounded by typical for α′-hexagonal phase needles. A Ti-cubic structure was identified in this droplet-like shaped area. It is notable that this area was polycrystalline, and some dendritic structure was visible in the inner region. 

In pure Ti, Cu is known as a eutectic-forming β-stabilizing element. In Ti6Al4V alloy, V is another β-stabilizer. Therefore, it is reasonable to conclude that in the areas where concentration of V and Cu exceeded certain critical values, a high-temperature cubic β-Ti phase was stable down to the room temperature. Solubility limit of Cu in Ti6Al4V alloy, and critical transformation temperatures of Ti6Al4V-Cu alloys have not been systematically investigated yet. Present research showed that the α′-hexagonal phase in the rapidly cooled Ti6Al4V (ELI)-1 at % Cu alloy can dissolve up to about 5 wt % Cu. Nevertheless, it has to be taken into account that due to high cooling rates at LPBF, the diffusion could be suppressed. Therefore, the concentration of Cu observed in the α′-hexagonal martensite phase formed at cooling after LPBF can be higher than the equilibrium solubility limit of Cu in the conventionally manufactured Ti6Al4V-Cu alloy. 

Segregation of Cu at boundaries of the molten pool, and generally observed inhomogeneity of the LPBF manufactured Ti6Al4V (ELI)-1% Cu alloy, could be explained with respect to properties of the feedstock materials. The previous analysis [26] showed that the energy needed for melting of a unit volume of Ti6Al4V and Cu is very similar, 5.67 J/mm^3^ and 5.49 J/mm^3^ for Ti6Al4V and Cu respectively. Particles of Cu, due to the lower melting temperature were molten first, and then transferred heat to the surrounding titanium alloy [26]. It could explain, why unmolten pure Cu particles were not observed in the final material. Ti6Al4V powder particles were also eventually molten completely. Further homogenization, therefore, was going on in the liquid phase. Viscosity of the liquid Ti at temperatures of 1477–1777 °C is 4.42 mPa·s [27]. For the pure liquid copper viscosity changes from 4.03 mPa·s at 1263 °C to 1.96 mPa·s at 1677 °C [28]. At the same time, density of Cu is about twice higher than density of Ti alloy. If mass transfer by convection and mixing in the relatively viscous Ti alloy melt was not sufficient for the complete homogenization, due to lower viscosity and higher density Cu could sink into the melt and agglomerate near the molten pool boundaries. At the same time, mass transfer by diffusional processes in the liquid phase was sufficient to dissolve some Cu in Ti6Al4V. It can explain why areas with different concentration of Cu, but no areas of an unalloyed Ti6Al4V material, were observed in the final LPBF Ti6Al4V (ELI)-1 at % Cu material. Since the homogeneity of the molten pool mostly depends on the convectional processes and a time of the pool existence, application of the rescan strategy influenced the homogeneity positively. Nevertheless, application of the rescan strategy did not lead to the formation of a perfectly homogeneous solid solution. 

### 3.5. Mechanical Properties

Basic mechanical properties of the Ti6Al4V (ELI)-1 at % Cu were characterized to understand an influence of alloying on mechanical performance of the in situ alloyed material. As a reference, mechanical characteristics of LPBF Ti6Al4V manufactured with the same equipment were used. Hardness of 383 ± 13 HV_0.2_ in as-built LPBF Ti6Al4V samples was measured, while hardness of Ti6Al4V (ELI)-1 at % Cu measured in the present investigation was 456 ± 20 HV_0.2_. According to the statistical analysis made with the t-test (*p* < 0.001), an observed increase in the hardness was statistically significant. Strength characteristics showed the same trend. Ultimate tensile strength of 1243 ± 49 MPa of the as-built non-polished mini-samples produced from Ti6Al4V (ELI) has been reported in [17]. In situ alloying with Cu led to a substantial increase of the ultimate tensile strength. Values of 1550 ± 126 MPa were detected in the present investigation.

Strengthening of Cu-alloyed Ti6Al4V alloy could be explained by several mechanisms. Guo et al. [29] suggested that formation of an intermetallic phase in LPBF Ti6Al4V–x Cu alloys could result in the higher values of hardness and strength. Because of the intermetallic phase precipitations, some embrittlement of in situ LPBF Ti6Al4V-x Cu alloyed samples has been suggested. At the same time, a solid solution strengthening effect of Cu in Ti6Al4V alloy and a refinement of α′-martensitic phase, Figure 4, are the other possible factors influencing the observed increase in hardness and strength in the investigated LPBF Ti6Al4V (ELI)-1 at % Cu alloy.

Fracture surfaces of the tensile specimens were investigated by means of SEM to resolve the fracture mechanisms. Generally, fracture surfaces had distinct cup-and-cone shape. Central regions of the investigated fracture surfaces were typical for Ti6Al4V manufactured with LPBF. Micro-dimples, formation of voids, quasi-cleavage and microstructure-related features of fracture were observed in a fibrous zone at cup/cone surfaces [17,25].

**Figure 6:**
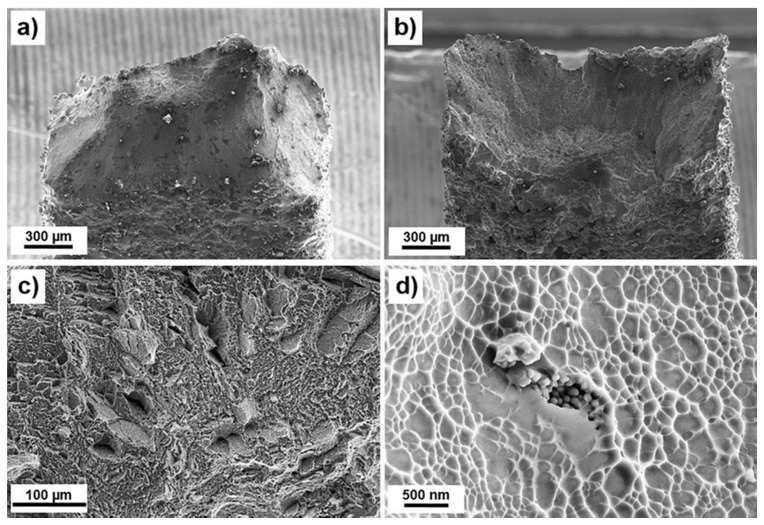
Fracture surface of LPBF Ti6Al4V (ELI)-1 at % Cu mini-samples: (**a**) and (**b**) opposite surfaces of a cup-and-cone fracture; (**c**) morphology of the fracture surface in the fibrous zone; and (**d**) area of the enhanced concentration of Cu observed at a shear lip.

Due to the high roughness in this region, the EDS analysis was difficult to perform. Nevertheless, in the investigated areas of the fracture surfaces, no correlation between fracture mechanisms and Cu content was observed. However, small Cu-enriched regions were observed at the shear lips, Figure 6, but it is difficult to confirm that they influenced material’s performance significantly. Otherwise, typical fracture surface with micro-dimples, sometimes elongated, was observed in this region.

**Figure 7:**
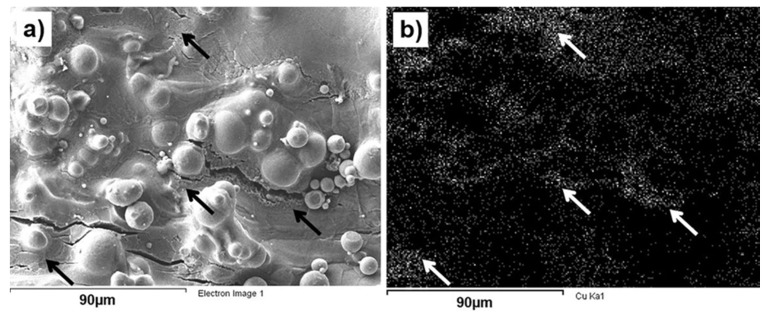
Side surface of LPBF Ti6Al4V (ELI)-1 at % Cu mini-sample nearby the fracture: (**a**) surface morphology; (**b**) CuK_α_ SEM-EDS map of the same region. Areas of high concentration of Cu marked with arrows.

As it is seen in Figure 6, a factor of surface roughness seems to be significant in the tensile tests of mini-samples. In all observed specimens, cup and cone were slightly asymmetric with a trend to move towards the edge. It could be a result of the mixed fracture mode accompanied with a nucleation and propagation of a crack from the surface due to stress concentration at surface roughness. Chemical analysis of the side surface adjacent to the neck and fracture showed that there were many small cracks in that region, Figure 7. Some of the cracks could be directly associated with Cu-rich areas at the surface, as indicated by arrows in Figure 7. Surface was too rough to get correct EDS signal and measure concentration of Cu in these regions, but according to the differences in morphologies between regions with different Cu content, those cracks were most likely nucleated in regions containing 20–35 wt % Cu. Nevertheless, fracture analysis showed that the fracture behavior was not due to mixed mode loading, which presented only at the edges. Unpolished mini-specimens investigated in this research could rather be interpreted as notch tensile specimens, with a notch shape controlled by surface quality. Therefore, tensile mechanical properties of LPBF Ti6Al4V-xCu obtained with the mini-samples are recommended to be verified with tensile test data obtained with standard specimens to investigate material properties, and notch strength ratio of LPBF manufactured materials. 

### 3.6. Antibacterial Functionalization

Manufacture of Ti6Al4V (ELI)-1 at % Cu alloys in situ by LPBF is a promising approach providing an opportunity to embed the added-value antibacterial performance directly to the custom implant in one step. Investigations have shown [29] that presence of the Cu resulted in a good antibacterial performance of the final alloy. Antibacterial tests [29] have demonstrated that alloyed Ti6Al4V alloys present strong and stable antibacterial property against *E. coli* and *S. aureus*. The best performance was observed for alloys with 4 and 6 wt % Cu. Additionally, the Cu-bearing alloys presented good cytocompatibility to the Bone Marrow Stromal Cells (BMSCs) from Sprague Dawley (SD) rats.

In the present investigation, growth of *E. coli* and *S. aureus* was monitored in the presence of sample discs. Bacterial suspensions (10^5^ CFU/ml) were applied directly to discs and viable counts determined after 24 h exposure. The presence of Cu in Ti6Al4V alloy discs resulted in a one order of magnitude decrease in the growth of *E. coli* and notable two order of magnitude decrease in growth of *S. aureus*, Table 2. Clearly, the growth of *S. aureus* was strongly influenced by the presence of Cu. Since copper ions kill bacteria by destroying the cell wall and cell membrane, causing the cytoplasm to leak [21], it is tempting to speculate that the cell membrane difference between Gram-negative *E. coli* and Gram-positive *S. aureus* could explain why *E. coli* was affected less by the presence of Cu than *S. aureus*. This strain of *E. coli* has previously been shown to demonstrate less sensitivity to copper nanoparticles compared to *S. aureus* [30].

## 4. Conclusions

Alloying of Ti6Al4V with Cu was performed in situ by LPBF from a mixture of Ti6Al4V (ELI) and 1 at % Cu powders. The produced material exhibited no solidification cracks. Complete melting of Cu particles was observed. 

Although both Cu and Ti6Al4V (ELI) particles were molten completely, segregation of Cu was observed on the molten pool boundaries. This effect was discussed in relation to differences in the viscosity and density of molten Cu and Ti6Al4V. 

The surface quality and more homogeneous surface distribution of Cu in the in situ alloyed Ti6Al4V (ELI)-1 at % Cu can be achieved by decreasing the scanning speed and hatch distance or applying the rescan manufacturing strategy.

In the bulk material, a complete alloy homogeneity was not achieved. Observed areas with higher Cu concentration were associated with fusion boundaries. Enrichment by Cu differed from area to area. It varied between 1–5 wt % in areas with α′-martensitic microstructure, and reached up to 20–35 wt % in areas with dendritic/cellular microstructure. The latter were identified as polycrystalline regions of β-Ti cubic alloy.

Strength characteristics measured with the tensile of mini-samples and hardness tests were higher than the values obtained for pure LPBF Ti6Al4V (ELI) alloy. Fracture surface was typical ductile cup-and-cone fracture, often reported for tensile fracture of LPBF Ti6Al4V. An influence of Cu-rich regions on changes in the fracture mechanisms in a fibrous zone was not observed. Nevertheless, nucleation of cracks at the surface was detected and associated with the regions with higher concentration of Cu. Presumably those areas were the areas with β-Ti cubic structure.

Antibacterial testing showed notable reduction in growth of pure cultures of *E. coli* and *S. aureus* at in situ alloyed LPBF Ti6Al4V (ELI)-1 at % Cu. It is necessary to conduct further testing not limited to only these two bacterial strains, but also emerging pathogens causing anaerobic infections associated with implants. Furthermore, it could be very useful to include isolates from failed implants, not only laboratory strains.

## Figures and Tables

**Table 1:** Surface roughness *R_a_*, µm and *R_z_*, µm, and the average concentration of Cu, wt % in in situ alloyed Ti6Al4V (ELI)-1 at % Cu single layers manufactured with single scan (S) and rescan (R) manufacturing strategy.

Scanning Speed	*R_a_*, µm/*R_z_*, µm/Cu, wt % at Hatch Distance			
	70 µm	80 µm	90 µm	100 µm
0.7 m/s, (S)	5 ± 0.7/25 ± 2.4/1.05	5 ± 0.1/34 ± 4.4/1.1	7 ± 0.7/38 ± 1.8/2.17	7 ± 0.4/46 ± 1.5/1.76
1.0 m/s, (S)	6 ± 0.6/39 ± 0.6/1.54	7 ± 0.6/42 ± 2.7/2.13	8 ± 1.0/44 ± 1.9/2.62	9 ± 0.2/51 ± 3.2/2.57
1.3 m/s, (S)	7 ± 0.8/38 ± 0.6/2.13	7 ± 0.7/45 ± 4.3/2.36	8 ± 0.6/51 ± 1.8/2.66	9 ± 0.3/54 ± 3.9/2.31
0.7 m/s, (R)	4 ± 0.1/25 ± 1.1/1.27	5 ± 0.7/27 ± 2.7/1.3	6 ± 0.3/36 ± 1.3/1.66	7 ± 1.2/40 ± 4.4/1.84
1.0 m/s, (R)	6 ± 0.9/36 ± 2.2/1.72	6 ± 0.9/36 ± 0.6/1.78	7 ± 1.4/40 ± 2.2/1.34	8 ± 0.5/51 ± 1.4/2.69
1.3 m/s, (R)	7 ± 0.3/37 ± 1.1/1.65	7 ± 0.5/37 ± 1.8/1.26	9 ± 0.5/50 ± 4.8/1.61	9 ± 1.0/51 ± 5.1/2.25

**Table 2:** Growth *E. coli* and *S. aureus* demonstrated after 24 h in the presence of Ti6Al4V and Ti6Al4V-1 at % Cu discs.

Bacteria	Colony Forming Units, (CFU)/mL		
	No disc	Ti6Al4V	Ti6Al4V (ELI) + 1 at % Cu
*E. coli*	1.48 × 10^9^	1.50 × 10^9^	1.27 × 10^8^
*S. aureus*	8.46 × 10^8^	2.24 × 10^8^	1.22 × 10^6^
